# Supplementary material for: Primary school staff perspectives of school closures due to COVID-19, experiences of schools reopening and recommendations for the future: A qualitative survey in Wales
Source: PLoS One. 2021 Dec 2;16(12):e0260396. doi: 10.1371/journal.pone.0260396 (PMC8639092; doi:10.1371/journal.pone.0260396)
Supplement: Attachment — Submitted filename: Response to Reviewers.docx [file pone.0260396.s002.docx]

Dear Dr Webster,

On behalf of all authors, we would like to thank both reviewers for taking their time to read our revised manuscript. Reviewer #2 was happy that we addressed their comments. We would like to extend our appreciation to Reviewer #1 who provided detailed feedback and comments in a second round of review of our manuscript. We feel this added input has strengthened the manuscript, particularly in relation to providing detailed information about the study’s theoretical underpinning, and improving the clarity of the findings with an additional summary, summarising key findings at the start of each sub-section. Please find below our response to reviewer #1, outlining changes made and the associated page/line numbers within the tracked changes submission for cross-reference. Furthermore, we have made some minor amendments to wording and included some relevant new literature that has been published since the original submission (e.g. [82] Sundaram et al 2021, page 37, line 920; and [65] Watermeyer et al 2021, page 31, line 776).

1.1 **Reviewer #1: Thank your responding to my recommendations. I do however still have a few concerns. My main concern is the lack of theoretical underpinnings --you are presenting the findings as empirical data yet your are seeking to reflect 'perspectives' of the teachers in a school. You slipped in the idea of interpretative approach to your study. My suggestion here is that you need to unpack interpretivism as your theoretical framing --both in regard to how you did your coding/analysis but also in how it was used to inform/shape the inferences drawn in your discussion. You need to tell the reader why this approach was most appropriate to your study and how it was used. This could go around line 81. After this you can add your section on aim of your study.** **Finish with your research question.**

Thank you for raising this important point regarding the theoretical underpinning of this study. We agree that offering a rich description of our theoretical approach would help the reader to interpret the findings with a greater degree of understanding. To address your suggestion of unpicking the interpretative approach, we have included additional theoretical information, including the use of a grounded theory approach in order to develop the recommendations in which our findings are based on. We now feel that we have justified our theoretical standpoint in relation to the framing of the study, coding, analysis and in informing the inferences from our study. Please find below specific revisions and page numbers to address your comment.

Introduction

- Grounded theory is a useful approach when little is known about a phenomenon[27]. In this case of this study, the rapidly evolving impacts of the COVID-19 pandemic brought with it unprecedented changes to society including national school closures based on limited evidence of potential challenges facing schools and learners. This study uses an interpretive approach with tenets of grounded theory methodology in order to develop a pattern of meanings, explanations and descriptions that reflect the perspectives and experiences of primary school staff within the context of school closures and phased reopening of schools. The purpose of the theoretical approach used in this study is to explore and extract the different perspectives in order to develop a rigorous and robust foundation for shaping and influencing emerging policy and practice in post-COVID education provision. Thus, the pragmatic approach to grounded theory in assessing and addressing challenges in practice [28] is suitably aligned to the aims of this study.

The aim of this grounded theory qualitative study is therefore to reflect on the perspectives of primary school staff regarding school closures between March and June 2020 and their experiences of the initial phased reopening of schools between June and July 2020 to identify recommendations for the full-time reopening of schools from September 2020 or future scenarios of school closures and blended learning **(page 4, line 91-117).**

Methods

- The basis of this study relies on an interpretivist approach, whereby the authors assume social reality is shaped by human experiences (primary school staff) and social contexts (primary school closures). Grounded theory research gathers data relating to participants’ different lived experiences in order to generate plausible theory to understand the contextual reality of common circumstances[26]. A grounded theory approach was applied to the data collection, analysis and explanatory findings in order to offer descriptions of the perspectives and experiences of primary school staff during the period of national school closures (between March and June 2020) and the phased reopening of schools (June to July 2020).

Generating theory in grounded theory relates to plausible relationships proposed amongst concepts and sets of concepts[29]. In the case of the current study, the generation of theory relates to the development of recommendations regarding the full-time return from September 2020, based on these lived experiences of primary school staff. Therefore, this approach intends to inductively construct these recommendations aligned to these lived experiences to contribute to the limited evidence base of the impact of school closures and to shape and influence emerging policy and practice during the ‘education recovery’ period following the COVID-19 pandemic. **(page 5, line 122-139)**

Methods – School staff survey

- An online survey with primary school staff was distributed in July 2020 through the HAPPEN primary school network (Health and Attainment of Pupils in a Primary Education Network) [30]. The survey was open for responses between 9^th^ and 31^st^ July 2020. Inclusion criteria for participating in the survey was any primary school staff member (headteachers, deputy headteachers, teachers, teaching assistants, support staff) working within a local authority maintained primary school in Wales, UK. A grounded theory strategy using thematic analysis was used to generate themes of recommendations based on survey responses to gain an understanding into the perspectives and experiences of primary school staff during the period of school closures (March to July 2020) and the phased reopening of schools (June to July 2020) to inform the full-time return to education from September 2020. **(page 6, line 141-150)**
- Given that interpretivist grounded theory is based on inductively developing patterns of meaning, a range of broad, open-ended questions were included to explore the research question. As grounded theory presumes limited preconceived ideas of the research topic, the survey incorporated polarised open-ended questions regarding school closures **(page 6, line 184-187)**

Methods – Data analysis

- This research study adopted an interpretivist approach through thematic analysis of open-ended survey responses. Thematic analysis is considered a suitable approach in gaining meaningful comprehension of patterns in participant perspectives captured using a range of qualitative methods including open-ended survey questions [35,36]. Thus, this study used thematic analysis as a data analysis technique to elicit participants’ perspectives and experiences relating to school closures and the initial phased reopening of schools. In providing context-specific explanations and descriptions of the impacts of school closures, this study used an overarching grounded theory methodology to inductively develop theory presented as recommendations to inform the full-time return to education and emerging policy and practice. Thematic analysis as an analytical approach in grounded theory studies has been used widely in other settings-based research [28]. **(page 9, line 239-255)**
- This stage incorporated a grounded theory approach whereby both researchers revisited the coded survey responses and followed a constant comparative method to progressively categorise and refine the themes and sub-themes. **(page 10, line 275-279)**
  1.2 **In the section on the aim I would like to see an explicit stated link back to your introduction --e.g., why was it necessary to reflect on the perspectives of primary school staff - simply to make recommendation is not enough --this is where you talk about our gap in understanding the conditions and implications of shut down and COVID on schools --this needs to be make clear.**

Thank you for this suggestion, we agree that an explicit stated link between the aim of our research study in addressing a gap in understanding of the implications of school closures would be clearer for the reader. Please find details below of this revision:

- The implementation of national school closures as a public health measure to reduce social contacts resulted in a range of unintended consequences. Evidence demonstrates the impact on children’s development, physical and mental health, social and economic outcomes and widening inequalities [8–12,14,15]. However, there is limited evidence regarding the challenges posed to schools during school closures and reopening, and a gap in understanding of the strategies for schools returning based on these impacts [22]. This study therefore aimed to address this gap in evidence. **(page 4, line 91-97)**
  1.3 **I would also like to see you change background to introduction.**

Thank you, we have amended this.

1.4 **In the results I still feel as if I am getting lost in the quotes - I suggest you start with either a figure/diagram/table or summary para --where you outline the key findings overall.**

We have addressed this useful point by incorporating a summary paragraph at the beginning of each findings sub-heading:

Prioritise the health and wellbeing of pupils and staff

- A key priority highlighted by school staff was the importance of prioritising the health and wellbeing of pupils and staff. In summary, school staff consistently mentioned a range of negative impact of school closures on children. This included physical health concerns such as decreased physical activity and fitness, weight gain and lethargy, mental health concerns including increased anxiety and lower wellbeing, and the impact on social development such as a lack of social interaction, excessive gaming and social media use. Additional challenges were also highlighted regarding children with additional learning needs (ALN). The impact on school staff wellbeing included challenges with work/life balance such as the pressure of providing online teaching and learning whilst balancing personal issues and family life. Therefore, the recommendation to prioritise the health and wellbeing of pupils and staff included suggestions of specific wellbeing activities for pupils, ensuring there are designated staff responsible for wellbeing, protecting staff breaks and increased provision of outdoor learning and play. This was also emphasised in relation to staff perceptions that the return of face-to-face education would prioritise the rhetoric of “*catching up*” through intensive teaching and learning at the detriment of whole-school health and wellbeing. This will be discussed in more detail below. **(page 11, line 297-320)**

Focus on enabling parental support and engagement

- The importance of the home learning environment during school closures was highlighted as a key factor in children’s learning progression. This is outlined in the findings from this recommendation theme in which primary school staff noted widening inequities and inequalities. Staff in this study commented that the large variation in home learning environments and parental support/engagement accounted for gaps in children’s learning. This theme encompasses aspects of parental support and engagement that are both positive; one-to-one support, wider skill development, improved parent/school relationships, parental awareness of children’s learning needs, and negative; key skill regression, lack of engagement with/decreased motivation for learning. School staff acknowledged a wide range of barriers for parents and families supporting home learning, including working commitments and language barriers. Therefore, staff suggested introducing support systems such as advice helplines for parents, and emphasised the importance of strengthening links between schools and families. **(page 16, line 425-438)**

Enhance (pupil, parent and staff) digital competence through increased access and support

- During lockdown and school closures, teaching was primarily delivered through digital methods requiring children to engage in remote learning online. Also associated with children’s learning progression and gaps in learning, this recommendation theme covers a range of digital barriers. Pupils experienced digital exclusion through a lack of access to online learning materials and digital equipment (e.g. internet access, laptop) and competing demands for equipment in the household. Staff in this study also highlighted that digital competency was an issue for pupils, teachers and parents, with a lack of digital understanding hindering pupils’ ability to learn, teachers’ confidence and ability to teach online and parents’ ability to support their child with online home learning. Whilst staff suggested the provision of digital equipment and internet access, they emphasised that this should be matched with digital training for all, including teacher training for blended learning and training to support children and parents accessing online learning materials. This will be discussed in more detail below. **(page 20, line 514-527)**

Adapt the learning environment and teaching practice

- This recommendation theme represents school staff experiences during the phased reopening of schools, including adaptations to regular teaching practice (e.g. smaller class sizes) and the learning environment (e.g. higher staff to pupil ratio) that benefitted pupils. Staff in this study suggested that decreasing class sizes and increasing staff numbers would support whole-school wellbeing and allow learning support targeted to need. However, staff acknowledged that this required significant government investment and support to facilitate longer term adaptations to education provision. **(page 23, line 578-584)**

Clear communication of guidance and expectations

- This final theme constitutes the importance of clear and regular communication between government and schools, the possibility of schools receiving advance notice of changes in guidance to support planning and expectations, and the challenges of incorporating generic guidance to fit within the contextual school differences and needs. **(page 25, line 636-639)**
  1.5 **I would remove the word 'recommendation' from the key finding headings in the finding section and leave this term/terminology to the discussion. Otherwise it starts to sound like a report and not an academic paper.**

We have amended this suggestion and agree that it is in line with the presentation of an academic paper.

1.6 **I would remove dot points from conclusion - write as a narrative.**

Thank you for this suggestion. We have addressed this by presenting the conclusion as a narrative discussion of the recommendations, removing dot points. **(page 39)**

1.7 **I am not familiar with the references style used? the use of dot points and numbers in the references list is this correct??**

This paper uses the Vancouver referencing style, in line with the requirements by PLOS. This uses dot points in-text and numbers in the reference list:

PLOS uses the reference style outlined by the International Committee of Medical Journal Editors (ICMJE), also referred to as the “Vancouver” style. Example formats are listed below. Additional examples are in the [ICMJE sample references](http://www.nlm.nih.gov/bsd/uniform_requirements.html).

<https://journals.plos.org/plosone/s/submission-guidelines#loc-references>

1.8 **I do believe you have a good study with some important information ---the use of theoretical underpinning and a tightening up in the presentation of the findings for clarity would enhance this paper.**

Thank you for this positive comment. We appreciate the time you have committed to offering a second round of revisions for our paper. We feel we have addressed your concerns, in particular regarding the theoretical underpinning of our study and believe this has significantly strengthened the manuscript. We have also provided a summary paragraph at the start of each of the findings sub-headings to ensure clarity for the reader. We also believe that the use of in-text narrative quotes will be clearer for the reader when the manuscript is typeset.

**2.1 Reviewer #2: Thank you for a further opportunity to review the manuscript. I think the authors have clearly and thoughtfully addressed my comments and am happy with their revisions.**

Thank you.

Thank you again for the opportunity to submit a revised version of our manuscript addressing reviewers comments. We look forward to hearing from you.

Best wishes,

Dr Emily Marchant
