# Supplementary material for: Primary school staff perspectives of school closures due to COVID-19, experiences of schools reopening and recommendations for the future: A qualitative survey in Wales
Source: PLoS One. 2021 Dec 2;16(12):e0260396. doi: 10.1371/journal.pone.0260396 (PMC8639092; doi:10.1371/journal.pone.0260396)
Supplement: Attachment — Submitted filename: Response to Reviewers.docx [file pone.0260396.s003.docx]

Dear Dr Webster,

On behalf of all authors, we would like to again thank both reviewers for taking their time to read and comment on our revised manuscript.

**Reviewer #1:**

1. **Thank you so much for responding to my suggestions. I am pleased with the revisions which have improved the readability of this paper. This is important research which will offer some useful recommendations to the school system in how to operate in the COVID environment and the post COVID-'normal'.**

**I have one suggestion that I did make prior which I encourage you to consider and that is the inclusion of a stated central research question (i.e., line 83). As you are now using a grounded study to guide this work--you could start with: e.g. What were the challenges posed to schools during school closure and re-opening and how can the impact of these challenges be addressed going forward?**

Thank you again for reviewing the second revised manuscript. We are glad that you are satisfied that we have addressed the useful comments provided in the second round of revisions. We agree that a central research question would be important and have included this in line 83 as suggested.

Introduction. Page 4, line 86-89: This study therefore aims to address this gap in evidence by exploring what were the challenges posed to schools during school closures and reopening and how can the impact of these challenges be addressed going forward.

**Reviewer #3:**

2. **I read the revised version of the paper and I can see that the authors took great care in responding addressing to the reviewer comments. As I see it, the paper has still a number of issues that remain critical. My greatest concern is that the paper reads (still) too much like a policy report and not enough like an academic paper. Of course this has to do with the fact, that authors provide a whole extensive policy recommendations (but that might me a question of taste). The second issue relates to the quality of the convenience sample. I will elaborate on these issues below.**

Thank you for providing feedback regarding this second revised manuscript. Our manuscript explores the central research question of what were the challenges of school closures and reopening and how can the impact of these challenges be addressed going forward. We conducted this research as a rapid qualitative study to gather the experiences of a range of primary school staff in Wales, UK during the period school closures and reopening in 2020. Our methodological approach and discussion of findings aims to address this research question most suitably in the context of the COVID-19 pandemic.

Both the immediacy and uncertainty of the COVID-19 situation influenced our study design and theoretical underpinning, using a grounded theory approach to generate an understanding of the key challenges experienced by education practitioners in Wales as a result of national school closures. During this period and over the coming months and years, there are rapidly evolving strategies and policies developed by the government and national education bodies aiming to combat some of these challenges. These policies directly impact education practitioners in Wales, and their approaches to teaching in learning in the context of ‘education recovery’. The emergency nature of the COVID-19 pandemic and its impact on educational settings requires a research approach that contributes evidence in a manner that translates findings than can inform policy and practice that impact the education workforce.

We acknowledge your comment that the manuscript may read in certain places as a policy report. We feel that the presentation of our study findings in the manner of a research/policy/practice paper is hugely important during the COVID-19 pandemic, a period which requires rapid reporting of evidence and data to inform emerging policy and practice. This is supported in a recent paper by Vindrola-Padros et al. (2020), ‘Carrying Out Rapid Qualitative Research During a Pandemic: Emerging Lessons From COVID-19’. The authors reflect on their experiences of conducting rapid qualitative research during COVID-19, and suggest the importance of translating and sharing evidence as “*actionable findings*”. The authors state that “*this refers to straightforward recommendations that can be easily understood and translated into changes in policy and/or practice and requires carefully planning the use of findings during the research design phase. Even if qualitative studies are produced during epidemics, public health officials might have difficulties trusting the findings, digesting the information and translating it into changes in policy and practice*” (page 2193).

Conclusion: Page 39, Line 907-909: This work, along with related findings, has contributed to emerging policy and practice at the national scale in Wales, offering policymakers an evidence base for school-based practice into 2021 and beyond.

To acknowledge your comment regarding the readability of the manuscript, we have included an additional explanation of why we have structured the manuscript as a set of recommendations, citing the interesting paper by Vindrola-Padros et al. (2020):

Introduction. Page: 3, Line 59-62: Furthermore, it is important for rapid research conducted during the COVID-19 pandemic to be shared as “*actionable findings*”, recognised by Vindrola-Padros and colleagues as “*straightforward recommendations that can be easily understood and translated into changes in policy and/or practice”* [22,23]*.*

Page: 5, Line 97-99: The purpose of the theoretical approach used in this study is to explore and extract the different perspectives in order to develop a rigorous and robust foundation of “*actionable findings*” [24] for shaping and influencing emerging policy and practice in post-COVID education provision.

Methods. Page 6, Line 122-125: In the case of the current study, the generation of theory relates to the development of recommendations, viewed as “*actionable findings*” [24] regarding the face-to-face return from September 2020, based on these lived experiences of primary school staff.

Discussion. Page: 28, Line 637-638: Furthermore, this needs to acknowledge the challenges posed during reopening and develop strategies based on “*actionable findings*” as to how countries can return to a “*new (ab)normal*”, informing emerging policy and practice [24,45].

Conclusion. Page: 39, Line 904-907: It is essential for research to focus on the mechanisms that optimise and improve outcomes for children both during and after the COVID-19 pandemic, and translate these as “*actionable findings*” to inform emerging policy and practice [21,24].

Vindrola-Padros C, Chisnall G, Cooper S, Dowrick A, Djellouli N, Symmons SM, Martin S, Singleton G, Vanderslott S, Vera N, Johnson GA. (2020) Carrying Out Rapid Qualitative Research During a Pandemic: Emerging Lessons From COVID-19. Qual Health Res. Dec;30(14):2192-2204. doi: 10.1177/1049732320951526.

3. ***Theoretical underpinning***

***A main issue in the previous round of reviews was the question of the theoretical underpinning of the study. As suggested by reviewer #1, the authors now provide further arguments for why the chosen interpretative/grounded theory approach is suitable (mostly related to the newness of Covid-19). While I agree with that premise, the fact that so many heterogenous topics are discussed (student learning, student well-being, digital learning) leads to a somewhat superficial treatment of each of them, respectively. This might be a less severe problem, if the research design would have been able to estimates of the prevalence of the different issues across as the entire population of school teachers for the selected grades - but that would have required a sufficiently large random sample of this population). In any case – I find that the manuscript still lacks theoretical depth.***

Thank you for your comments regarding the theoretical underpinning of our study. It is important to acknowledge that this manuscript has already received two rounds of revisions from two previous reviewers. During these stages of revisions, reviewer #1 raised a number of suggestions regarding additional justification and explanation of the interpretative/grounded theory approach. We are pleased that reviewer #1 is satisfied with these revisions and feels we have adequately addressed issues regarding the theoretical underpinning, including our justification of this approach particularly in the context of COVID-19.

The use of an interpretative/grounded theory approach enabled the perspectives of education practitioners to be captured and conceptualised as key recommendations aligned to challenges and impacts of school closures and reopening. In line with the grounded theory approach which presumes limited preconceived ideas of the research topic, the survey incorporated open-ended questions regarding school closures (e.g. benefits/negative impacts of school closures, concerns and recommendations for face-to-face reopening). This was in order to gather information rich narratives of participants’ perspectives, experiences and recommendations to address the central research question.

We value your comments regarding the topics discussed in this manuscript and that the broadness of themes captured can be considered heterogenous. The broad and exploratory nature of the open-ended questions, in line with the grounded theory approach, allowed the wide range of impacts and challenges to be captured from those at the frontline of education provision. We have included verbatim quotes in order to illustrate the thematic descriptions. Our findings have been validated by academic research, reports and policy briefs published in the interim of this review period, including at a global level by UNESCO and the WHO. This also includes extensive research within the UK by the National Foundation of Educational Research (NFER) conducted during the COVID-19 pandemic. Findings from the current study are mirrored within a number of reports and policy briefs, with topics such as pupil engagement with learning, impacts on learning and academic outcomes, health and wellbeing, digital barriers, pressures on the education workforce and widening inequalities discussed synchronously (Sharp et al. 2020, Nelson and Sharp 2020, Nelson et al. 2021, Rose et al 2021). We are confident that our grounded theory approach has encapsulated the wide range of impacts reported by school staff and in agreement with the wider literature published since submission.

Whilst the findings in the current study cover the wide ranging impacts of school closures, we feel this provides a solid foundation for further research to be conducted exploring these topics in greater detail, particularly as we emerge from the period of school closures to the return of face-to-face teaching and learning.

We have included an additional suggestion of future research to explore these topics in greater detail within the conclusion:

Conclusion. Page: 40, Line 924-927: The broad nature of these recommendations cover the wide ranging impacts of school closures. This calls for future mixed-methods research to explore each topic in greater detail to examine and quantify the depths of these impacts and challenges, particularly since the return of face-to-face education provision.

In relation to your comment regarding the research design and sampling strategy, we feel that estimating the prevalence of issues amongst an entire population of school teachers requiring a large sample would call for a different study that would suit a quantitative design. Sample sizes and prevalence rates are at odds with the premise of qualitative research, which aims to explore the rich lived experiences of participants, as opposed to quantifying prevalence of experiences. In this study, and in line with qualitative research approaches, we were not aiming to be representative/generalisable of the population, and have not claimed this was our approach within the manuscript. Rather, our study aimed to capture and explore the lived experiences of the education workforce in relation to an understudied topic during an emergency period of national school closures. Convenience sampling was most suitable approach during this challenging period for the education workforce and was required in order to conduct rapid qualitative research. As noted above, we have advocated for further research to quantify these impacts. We will continue addressing your comments regarding the sampling strategy in the sub-section below.

Sharp C, Nelson J,Lucas M, Julius J, McCrone T and Sims D (2020) The challenges facing schools and pupils in September 2020. NFER. Slough. Available at: <https://www.nfer.ac.uk/schools-responses-to-covid-19-the-challenges-facing-schools-and-pupils-in-september-2020/>

Nelson J and Sharp C (2020) Schools' responses to Covid-19: Key findings from the Wave 1 survey. NFER. Slough. Available at: <https://www.nfer.ac.uk/schools-responses-to-covid-19-key-findings-from-the-wave-1-survey/>

Nelson J, Lynch S and Sharp C. (2021) Recovery During a Pandemic: the ongoing Impacts of Covid-19 on Schools Serving Deprived Communities. NFER. Slough. Available at: <https://www.nfer.ac.uk/recovery-during-a-pandemic-the-ongoing-impacts-of-covid-19-on-schools-serving-deprived-communities/>

Rose S, Twist L, Lord P, Rutt S, Badr K, Hope C and Styles B (2021) Impact of school closures and subsequent support strategies on attainment and socio-emotional wellbeing in Key Stage 1: Interim Paper 1. NFER. Slough. Available at: <https://www.nfer.ac.uk/impact-of-school-closures-and-subsequent-support-strategies-on-attainment-and-socio-emotional-wellbeing/>

3. ***The empirical foundation
I am in serious doubt to what extent the research design which is based on a convenience sample realized with snowball sampling and similar methods can really be used to “develop a rigorous and robust foundation for shaping and influencing emerging policy and practice in post-Covid education provision” – as argued by the authors. While the identified topics and different perspective identified in the paper are very interesting, no doubt, I wonder whether a different and more systematic sampling approach (either qualitative or quantitative) would have led to very different results. Or - in other words, there is a realistic chance that the teachers who participated in the study might a quite different (special) – and had a very different experience of the Covid-19 pandemic and school closures – than the rest of the teacher population. As a minimum, the authors would need to address this issue in the paper.***

The method of convenience sampling was the most suitable approach to recruit participants during a global pandemic, recognising the breadth of lived experiences. We used a number of recruitment methods in order to increase the scope of the survey reach, and we have outlined the three stage approach used for disseminating the survey. The additional layer of snowball sampling aimed to increase the reach of our research study by recruiting school staff through key stakeholders in education, including regional education bodies, who have established relationships with a range of primary schools across Wales. Furthermore, recruitment was facilitated through the HAPPEN Wales primary school network which has existing partnerships with primary school across all local authorities in Wales, serving a wide range of deprivation areas and area characteristics. We are confident that we have accurately and transparently reported our sampling strategy within the manuscript.

You draw attention to an interesting point regarding the potential of a different sampling strategy yielding different results. It is indeed possible that those schools engaged with HAPPEN, regional education groups and those that participated in the survey could be considered as ‘research engaged’. This remains a challenge in all school-based research, and particularly during a time in which the education workforce was experiencing significantly higher working demands and increased stress. Perhaps most usefully, many of these schools and staff that participated in the study are also likely to be engaged with emerging policy design, for example the new *Curriculum for Wales* due to be rolled out in 2022, which has received significant input from ‘pioneer schools’ across Wales to its design and development. We aim to further extend collaborations through the HAPPEN network for future research projects to engage with wider groups and capture perspectives from those harder to reach.

Whilst we acknowledge your comment regarding different sampling strategies, we are still confident that the convenience and snowball strategy employed in our study is reflective of the perspectives of key education practitioners. Furthermore, our findings have since been validated in wider research across the UK since original submission (Sharp et al. 2020, Nelson and Sharp 2020, Nelson et al. 2021, Rose et al. 2021). Qualitative research allows an exploration of people’s experiences of education systems and policies including school closures and policy regarding the COVID response. This helps us to understand how these responses/policies can be shaped. We believe our actionable findings contribute to this understanding and have captured the perspectives of the education workforce in agreement with other literature.

To address your comments regarding sampling, we have included additional discussions in the limitations section acknowledging the challenges of convenience sampling in reporting findings, and that those that did not participate may have experienced the period of school closures differently and thus, reported different perspectives. We feel this is an important point to include and we are grateful that you have drawn this to our attention.

Strengths and limitations. Page 39, line 885-890: The perspectives captured in this study may not account for the full breadth of lived experiences of all primary school staff during school closures and reopening. It is possible that those that participated in this study work within schools that are ‘research engaged’. Those that did not participate may have different experiences and perspectives during this period to those reported in this study.

We look forward to hearing from you.

Best wishes,

Dr Emily Marchant
